# A Case Report of Autoimmune Encephalitis: Could Post-COVID-19 Autoimmunity Become a Lethal Health Issue?

**DOI:** 10.7759/cureus.25910

**Published:** 2022-06-13

**Authors:** Erinie Mekheal, Marina Mekheal, Sherif Roman, David Mikhael, Nader Mekheal, Rajapriya Manickam

**Affiliations:** 1 Internal Medicine, St Joseph's Regional Medical Center, Paterson, USA; 2 Internal Medicine, West Virginia School of Osteopathic Medicine, Lewisburg, USA; 3 Family Medicine, St Joseph’s University Medical Center, Paterson, USA; 4 Internal Medicine, St Joseph Hospital Medical Center, Paterson, USA; 5 Pulmoanry and Critical Care Medicine, St Joseph Regional Medical Center, Paterson, USA

**Keywords:** total plasma exchange, acute hemorrhagic leukoencephalitis, intravenous immunoglobulin, post covid-19 infection, autoimmune encephalitis

## Abstract

While it is primarily thought of as a respiratory illness, COVID-19 is now recognized as a multi-organ disease that can present with a wide range of clinical manifestations. Particularly in patients with severe respiratory illness, neurological manifestations ranging from headaches, and loss of smell to strokes have been associated with the virus. In the setting of resolving respiratory illness, it is important to consider autoimmune encephalitis (AE) in the instance of new-onset neurological manifestations. The typical patient presentation includes altered mental status, fever, seizures, and/or focal neurological deficits. These neurological manifestations make it crucial to consider either underlying COVID-19 infection or post COVID-19 autoimmunity so as not to delay the administration of the appropriate treatment. Herein, we present the case of an 88-year-old female with new-onset right leg weakness, and dysarthria, that progressively developed to altered mental status months after having respiratory symptoms of COVID-19. According to the criteria of AE diagnosis, the patient’s clinical course and work-up findings proved the diagnosis.

## Introduction

Autoimmune encephalitis (AE) is an immune-mediated inflammation of the brain, the meninges, spinal cord, and/or peripheral nervous system [1,2]. Although symptoms vary according to the anatomical localization of inflammation, there is significant symptom overlap between all antibodies and all forms of AE [3,4]. The cause of AE is unknown in >50% of cases. However, multiple well-known precipitating factors have been documented including exposure to certain bacteria and viruses and specific types of tumors with complicated paraneoplastic syndrome [5]. AE mostly presents with memory or cognitive behavioral deficits without fever or alteration in the level of consciousness [3]. In this report, we discuss the case of an 88-year-old right-handed female who presented with new-onset right-leg weakness and dysarthria two months after having respiratory symptoms of COVID-19. She was initially thought to have encephalopathy secondary to chronic hypoxia from the previous infection; however, the patient’s evolved clinical course, MRI and cerebral spinal fluid (CSF) findings proved consistent with a diagnosis of probable AE prompting appropriate medical management [3].

## Case presentation

This is an 88-year-old female with a medical history of hypertension who presented to our facility with new-onset right leg weakness and dysarthria last seen normally 72 hours ago. The patient reported having generalized weakness over the past two weeks; however, denied any fever, chills, cough, diarrhea, dysuria or recent travel. The patient was hospitalized for COVID-19 pneumonia two months ago and was treated with steroids, remdesivir and supplemental oxygen therapy. The patient did not receive the COVID-19 vaccination prior to the presentation.

**Figure 1:**
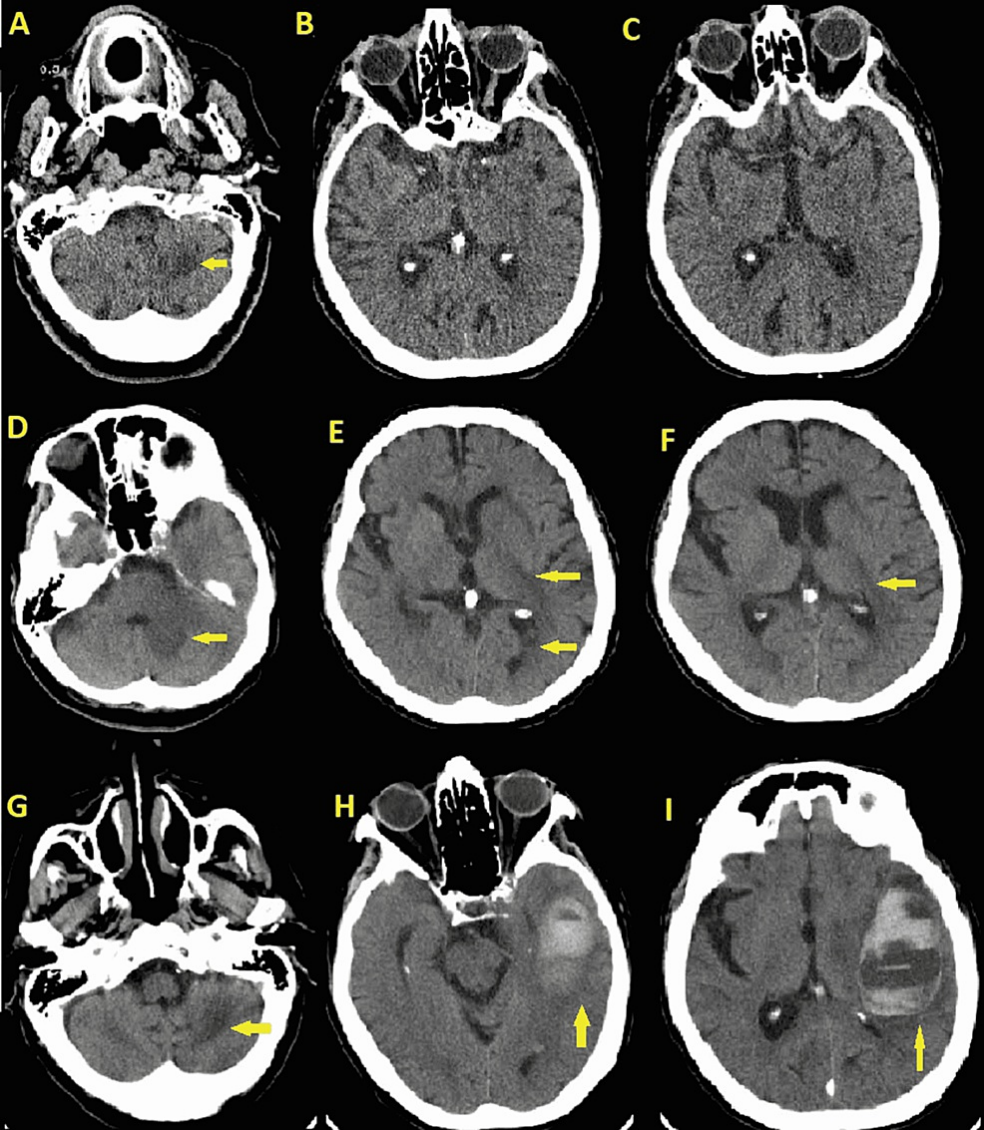
Computed tomography (CT) scan of the brain with/without contrast (A-C) CT brain stroke code on admission showing an old left cerebellar infarct (arrow in A). There is no evidence of acute infarct, hemorrhage, or extra-axial collection. (D-F) CT after 12 hours from admission showing an increasing hypodensity in the left temporal lobe (arrow in E, F) as well as within the left cerebellar hemisphere (arrow in D) consistent with evolving relatively acute infarcts. While there is no hemorrhage within these areas of evolving infarction although there is a small amount of blood within the left occipital horn (arrow in E). There is no developing midline shift although there is a mass effect on the fourth ventricle as a result of the evolving left cerebellar infarct. (G-I) Last CT before expiration showing a new temporal lobe intraparenchymal hematoma which measures approximately 5.9 x 3.8 cm (arrow in H, I) which results in mass effect on the left lateral ventricle and a resultant left to right midline shift of 5 mm. Small amount of blood within the temporal horn of the left lateral ventricle is consistent with intraventricular extension. No extra-axial collection identified. Hypodensity within the left cerebellar hemisphere (arrow in G) is similar to that of previous CT.

**Figure 2:**
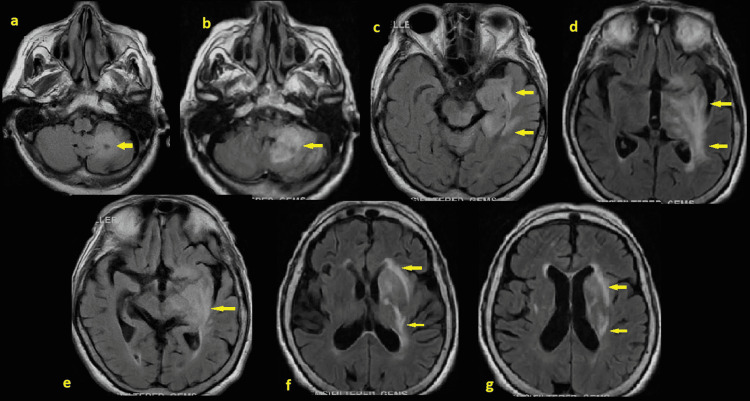
Magnetic resonance imaging of the brain on admission with/without contrast Magnetic resonance imaging (MRI) on admission showing an old infarct in the left cerebellum (arrow in a). There is a focus of restricted diffusion in the medial aspect of the left cerebellum consistent with an acute infarct (arrow in a, b).  There is edema surrounding this infarct. There is FLAIR hyperintensity within the margin of the cerebellopontine angle cistern, brachium pontis, left posterior midbrain (arrow in c), left cerebral peduncle, left medial aspect of the temporal lobe (arrow in d), left thalamus, left basal ganglia (arrow in e-g). There are areas of magnetic susceptibility within the left temporal lobe (arrow in d) and cerebellum (arrow in a) with associated edema causing mass effect with ill-defined somewhat low-level enhancement. The above could just represent relatively acute or subacute infarcts although infiltrating tumors or infectious/inflammatory processes cannot be entirely differentiated from the above. There is no evidence of definitive hemorrhage or extra-axial collection. There is asymmetric magnetic susceptibility within the left occipital horn, which may just represent more focal choroid plexus calcification although a small amount of associated hemorrhage cannot be excluded (arrow in d). Post-contrast imaging demonstrates slightly less vigorous enhancement within the medial temporal lobe and is in disparate of the arterial distribution. Findings have ruled out medial temporal lobe infarct, and presumably suggested a combination of vasogenic and cytotoxic edema from an infectious/inflammatory process and clinical correlation is requested. No definite mass is visualized. Mild non-specific white matter abnormality likely related to micro vascular disease. There is mild generalized parenchymal volume loss with no evidence for developing hydrocephalus, herniation patterns with midline shift.

On examination, she was afebrile, blood pressure was 178/79 mmHg, heart rate was 84 beats per minute, and oxygen saturation was 97% on room air. The patient had dysarthria with marked weakness of the right lower extremity and an NIHSS score of 9 was calculated. CT head and brain without contrast showed an old left cerebellar infarct with no evidence of acute infarct or hemorrhage (Figures 1A-1I). The patient was not a candidate for thrombolytic therapy or neurosurgical intervention due to the low NIHSS score and late time of presentation. Neurology scheduled her for an MRI with/without contrast and admitted her to the intensive care unit for acute ischemic left cerebellar infarct. She was initiated on antiplatelet and high-intensity statin therapy. Laboratory analysis was unremarkable. She had a negative SARS-CoV-2 PCR nasopharyngeal swab but a positive serum COVID-19 IgG antibody. Her electrocardiogram (EKG), and transthoracic echocardiogram were unremarkable. Within 12 hours, the patient developed altered mental status. Repeated CT without contrast showed an acute left cerebellar hemisphere infarct and questionable increasing hypodensity in the left temporal lobe concerning an evolving acute infarct and a minimal amount of blood within the left occipital horn. MRI with and without contrast results showed evidence of an old infarct, acute infarct involving the left cerebellum, as well as an effacement of the left temporal horn and edema within the left pons, midbrain, left temporal lobe and surrounding the basal ganglia. As per the radiology report, these areas are associated with some ill-defined low-level enhancement which could indicate infarcts with associated enhancement although an infiltrating tumor/infectious or inflammatory process cannot be entirely excluded. In addition, the questionable sign of hemorrhage within the left occipital horn may just represent a focal choroid plexus calcification although a small amount of associated hemorrhage cannot be excluded and clinical follow-up was recommended (Figures 2a-2g). 

Neurology, neurosurgery, and radiology have reviewed the images and concluded that the abnormal signals in the above-mentioned areas are disparate of their arterial distributions and not consistent with any discrete infarcts, infiltrating tumor, or associated edema from the LT cerebellar infarct. Additionally, neither the small left cerebellar infarct nor the tiny left occipital horn intraventricular hemorrhage (IVH) explained her poor mentation and there is no evidence of hemorrhagic transformation (HT) that might explain IVH in this region. All of which point toward the possibility of an inflammatory/infectious process. Further investigations showed elevated ESR, CRP, and D-dimer while the serum autoimmune panel was negative. EEG showed moderate-severe diffuse encephalopathy without epileptiform discharges or seizures. CSF opening pressure was normal. CSF analyses were unremarkable except for significant pleocytosis and elevated protein. Meningitis/encephalitis panel by PCR were negative, including E. coli K1, H influenza, L monocytogenes, N meningitidis, S agalactiae, S pneumonia, Cytomegalovirus, Enterovirus, HSV 1, 2, H parechovirus, HZV, EBV, Cryptococcus, and common geographic arboviruses (Table 1). 

**Table 1:** Laboratory tests with normal reference values. CBC, complete blood count; WBC, white blood cells; CSF, cerebrospinal fluid analysis; RBCs, red blood cells.

Laboratory test	Results	Normal
CBC		
WBCs (x10^3^ cells/mm^3^)	10.8	4.5-11
Band cell (%)	0	0-10
Procalcitonin (ng/mL)	<0.05	<0.1
CSF		
appearance	colorless, clear appearance, non xanthochromic	Clear/colorless
Specific gravity	1008	1006-1007
Glucose (mg/dL)	75	50-75
Protein (mg/dL)	145	15-45
RBCs (cells/mm^3^)	53	0-5 cells
WBCs (cells/mm^3^)	15 (18% seg neutrophil, 66% lymphocytes, 16% monocytes)	0-5 cells
Gram stain	negative	Negative
Meningitis/encephalitis panel by polymerase chain reaction test	negative	Negative
Microbial culture	Negative	Negative

**Figure 3:**
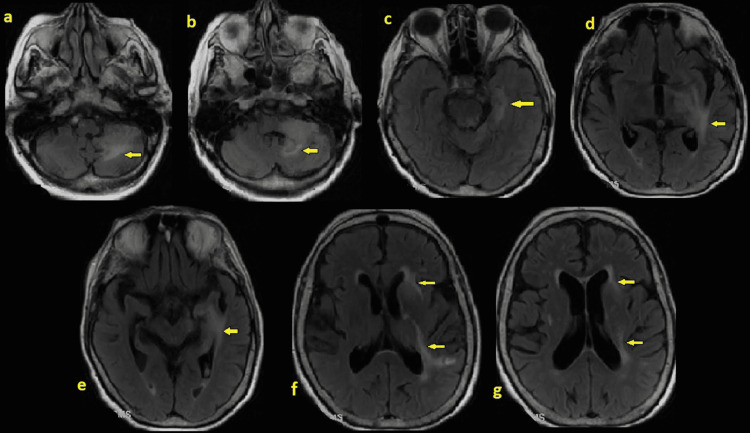
Repeated magnetic resonance imaging (MRI) of the brain with/without contrast after IVIG, and steroids. Magnetic resonance imaging (MRI) of the brain after IVIG, and steroids showing no evidence of new infarct, areas of hemorrhage, or mass lesion. There are poorly defined areas of FLAIR hyperintensities involving the left cerebral peduncle, left cerebellum (arrow in a-c), left medial temporal lobe (arrow in d), basal ganglia (arrow in e, f) and periventricular white matter (arrow in f, g), which demonstrates slightly irregular enhancement after contrast administration as well as areas of restricted diffusion. Compared to prior brain MRI, there is a significant interval change with decreased enhancement within areas of abnormality mentioned above. Clinical correlation is requested. There is also an improving depended hemorrhage in the occipital horn of the left lateral ventricle. There is mild generalized parenchymal volume loss with no evidence for midline shift, obstructive hydrocephalus, or herniation pattern. There is volume loss. The normal intracranial flow voids are noted. The calvarium, sella turcica and cervicomedullary junction are unremarkable. There is no evidence for acute sinusitis or mastoiditis. The seventh and eighth neural vascular bundles are unremarkable.

Given the clinical presentation, laboratory, and imaging results, a diagnosis of AE was made. The patient was started on intravenous Methylprednisolone 250 mg every six hours for a five-day course with mild mental status improvement and minimal changes in repeated MRI. A decision was made to start Intravenous Immunoglobulin (IVIG) 0.4 g/kg/day x for a total of five days with a plan to hold off on tapering steroids until after IVIG is completed. Subsequently, the patient's mental status has been improved, and no longer requires ICU level care. Another repeated MRI showed improvement with poorly defined areas of FLAIR hyperintensities involving the same areas mentioned above (Figures 3a-3g). Eight days later, the patient developed a new-onset altered mental status. Repeated CT brain showed new left temporal intraparenchymal hemorrhage with midline shift and intraventricular extension (Figures 1A-1I). Neurosurgery was re-consulted and the patient was not found to be a good surgical candidate due to advanced age, location, and size of the bleed with overall poor prognosis for which the family pursued comfort measures.

## Discussion

While AE after SARS-CoV-2 in adults is thought to be rare, a study at Mayo Clinic Rochester found that 0.05% of 100,384 patients diagnosed and cared for with any COVID-19 illness in 2020 had AE [3]. These five patients met definite, probable, or possible AE diagnostic criteria [3]. In another study, 80% of the cases developed before or during the acute COVID-19 infection, while 20% of cases developed as post-infection sequelae. Therefore, our patient represents the small percentage of cases that develop post COVID-19 AE with a delayed onset of two months.

In some of the reported cases, there was a normal CSF protein, while other cases showed evidence of CSF pleocytosis. Some cases had negative autoantibody panels and others had positive results [6]. Moriguchi et al. reported a case in which the specific SARS-CoV-2 RNA was found in CSF with labs showing mononuclear pleocytosis. In this case, the patient had a negative nasopharyngeal swab for SARS-CoV-2 and had post-seizure neuroimaging that demonstrated hyperintensities in the medial temporal lobe, hippocampus, and lateral ventricle confirming COVID-19 post-infection AE [7]. Our case was found to have COVID-19 antibody in the serum, along with CSF pleocytosis, which indicates that COVID-19 is the culprit.

The exact mechanism of the post-COVID-19 infection inducing AE is still currently debated. However, multiple theories were reported in the literature review. The first is thought to be molecular mimicry with anti-neuronal autoantibodies that mark host autoantigens as foreign and induce CNS damage [6,8]. Unfortunately, the AE autoantibodies panel of our patient was not available. However, this theory emphasizes the importance of monitoring self-reactive antibodies and autoimmune responses during vaccination trials against COVID-19, especially in people who experienced post-COVID-19 AE, such as our patient. The second theory is excessive cytokine release causes damage to the CNS, which explains why our patient has elevated ESR, CRP, and D-dimer [6,9]. The last theory is reported to be due to hematogenous spread or direct introduction of the virus from the nasal cavity to the CNS [6]. However, our patient did not have any respiratory symptoms at the time of presentation, and she had a negative SARS-CoV-2 nasopharyngeal swab.

There are factors associated with increased risk of HT of ischemic stroke (HT), including stroke severity, reperfusion therapy (thrombolysis and thrombectomy), and NIHSS at presentation (NIHSS < 10 has <13% rate of HT), hypertension, hyperglycemia, and age [10]. Although our patient was old and was initially hypertensive, she did not receive thrombolytic therapy or any neurosurgical intervention. Her NIHSS was 9, was not hyperglycemic, and her blood pressure was under control throughout her entire hospital stay. Additionally, her bleeding was in the temporal area, away from the original ischemic infarct site. All of these raise the question of the role of her AE or previous COVID-19 infection or their combination in the development of intracranial hemorrhage (ICH).

A large retrospective, cross-sectional analysis of patients enrolled in the American Heart Association was recently conducted. It showed that compared to ischemic stroke, ICH is a much rarer complication among patients with current or previous COVID-19 infection [11]. The fact that anticoagulants (AC) were held for our patient has put her at lower risk of developing such complications. In the meantime, there have been a few cases of COVID-19 complicated by acute hemorrhagic leukoencephalitis (AHLE) [12]. AHLE is a life-threatening neurological syndrome that consists of progressive encephalopathy due to hemorrhagic lesions of the white matter. These lesions are characterized by polymorphonuclear infiltrates, small vessel necrosis, and overall demyelination [13]. Due to the low frequency of this disease, it is underreported, and diagnosis relies on clinical presentation, imaging, CSF analysis, and histopathology [14]. A patient who presents with rapid deterioration, and a subsequent coma, post-infection, should clue the physician on the possibility of AHLE and warrant a biopsy for diagnosis [13]. Our patient rapidly deteriorated with developing ICH which led us to interpret it as a possible life-threatening AHLE. Unfortunately, an autopsy was not pursued which would be the definitive method for confirming the diagnosis.

Although AE is a rare and depleting disease, it has a good prognosis. Early aggressive treatment has been shown to have better outcomes and fewer relapses [15]. First-line treatments include immunotherapeutic agents like corticosteroids, IVIGs, and total plasma exchange (TPE) [15]. Unfortunately, even with the early treatment of our patient, she had a poor outcome. This leads to an important question in clinical neuroscience. What factors correlate with the outcome of AE, and whether COVID-19 infection negatively affects the outcome, even with early, aggressive, and appropriate management. A multicenter study in eastern and central China was performed and concluded that, regardless of study limitations, several risk factors have been associated with a poor prognosis of AE [16,17]. These factors include older age, mental and behavioral disorders, movement disorders, impaired consciousness at admission, central respiratory depression, EEG with ≥50% slow waves, and tumors [16,17]. All of which explains why our patient's outcome was less favorable. However, further studies of these prognostic factors after the COVID-19 pandemic are required to guide physicians in the management of AE and improve the outcomes.

## Conclusions

AE following COVID-19 infection, while rare, should be considered with any case that presents with a combination of focal neurological, neuropsychiatric symptoms, and/or new-onset altered mental status, following resolution of respiratory symptoms. The under-recognized nature of the diagnosis is associated with higher mortality rates as early aggressive therapy is crucial to recovery. While AE responds to immunotherapy, further studies are required to investigate whether COVID-19 infection negatively affects treatment outcomes, even with early aggressive management. In addition, more studies are warranted to fully understand the mechanism of AE in cases of COVID-19 as well as monitoring for the development of post-challenge autoimmunity in COVID-19 vaccination trials.
